# Laparoscopy Prior to Laparotomy in Primary Debulking Surgery for Advanced Epithelial Ovarian Cancer in the U.S.: A National Cancer Database Analysis †

**DOI:** 10.3390/cancers18142195

**Published:** 2026-07-08

**Authors:** Nicole Goncalves, Kelly Lamiman, Michael Silver, Ioannis Alagkiozidis

**Affiliations:** 1 Department of Obstetrics & Gynecology, Maimonides Medical Center, 4803 10th Ave, Brooklyn, NY 11219, USA; 2 Division of Gynecologic Oncology, Department of Obstetrics & Gynecology, Maimonides Medical Center, 4803 10th Ave, Brooklyn, NY 11219, USAialagkiozidis@maimo.org (I.A.); 3 Department of Research Administration, Maimonides Medical Center, 4803 10th Ave, Brooklyn, NY 11219, USA; msilver@maimo.org

**Keywords:** epithelial ovarian cancer, primary debulking surgery, laparoscopy

## Abstract

**Objective:** Previous studies have demonstrated that the use of laparoscopy prior to laparotomy for primary debulking surgery (PDS) in advanced ovarian cancer is linked to lower rates of suboptimal cytoreduction. However, its impact on overall survival remains uncertain. This study aimed to compare overall survival and surgical outcomes between PDS cases initiated with laparoscopy and those without. **Methods:** We identified patients in the National Cancer Database who underwent PDS for stage IIIC and IV epithelial ovarian cancer between 1 January 2010 and 31 December 2017. Patients were divided into two groups for analysis: cases that were initiated with laparoscopy and those that were not initiated with laparoscopy. The primary outcome was overall survival, while secondary outcomes included 90-day postoperative mortality, 30-day readmission rate, and length of hospital stay. Statistical analyses were performed using SPSS version 29.0. **Results:** Among 12,532 eligible patients, 529 cases (4.2%) were initiated laparoscopically. Laparoscopy utilization increased from 3.4% to 6.9% over the study period. Rates of complete gross resection were similar between groups. Postoperative outcomes, including 90-day mortality, 30-day readmission, and hospital stay, did not differ significantly. However, laparoscopy was associated with improved overall survival in patients who proceeded to PDS (60.8 vs. 51.6 months, *p* = 0.012). In stage-stratified analysis, a survival benefit was noted in stage IIIC (64.5 vs. 55.2 months, *p* = 0.026), but not in stage IV cases. **Conclusions:** Despite its increasing use, the overall rate of laparoscopy before PDS remains low. Laparoscopy prior to laparotomy is associated with improved overall survival in patients with stage IIIC epithelial ovarian cancer undergoing PDS.

## Simple Summary

Epithelial ovarian cancer is a devasting disease that is often diagnosed at late stages (III and IV). The standard of care for advanced epithelial cancer is either primary debulking surgery with adjuvant chemotherapy or neoadjuvant chemotherapy with interval debulking in select patients not fit for upfront surgery. Laparoscopy has been proposed as a method to triage patients to either primary debulking surgery or neoadjuvant chemotherapy. The use of laparoscopy has been linked to decreased suboptimal cytoreduction; however, the true extent of its impact remains unknown. Using a large national database, this study aims to identify primary debulking cases that were initiated with laparoscopy from 2010–2017 and to evaluate overall survival and other post-operative outcomes.

## 1. Introduction

Epithelial ovarian cancer (EOC) remains the most lethal gynecologic malignancy, largely because of late-stage diagnosis due to asymptomatic progression in early stages [1]. More than half of patients present with advanced disease (stage III–IV), characterized by diffuse peritoneal dissemination, omental involvement, and, in some cases, distant metastases [1]. Despite advances in systemic therapies in the past two decades, including the incorporation of targeted agents such as PARP inhibitors and antiangiogenic therapies, long-term survival outcomes remain poor [2,3]. The five-year survival rate for advanced-stage disease is about 30%, which is only a modest improvement over the past 20 years. Additionally, the epidemiological burden of EOC remains significant, with an estimated 21,010 new cases in the United States in 2026 [1]. These sobering statistics underscore the importance of optimizing initial treatment strategies, particularly surgical management.

The current standard of care for advanced EOC consists of a combination of cytoreductive surgery and platinum-based chemotherapy. The goal of surgery is maximal tumor debulking, as residual disease volume following cytoreduction is one of the most significant prognostic factors influencing both progression-free and overall survival [4]. Optimal cytoreduction has historically been defined as residual tumor nodules less than 1 cm in size (R1), while complete gross resection (R0) indicates no visible residual disease. The relationship between optimal cytoreduction and survival was first established in the 1970s by Griffiths et al. and has been supported by numerous studies since [5]. Specifically, studies have demonstrated a stepwise improvement in survival outcomes with decreasing residual disease burden, with the greatest benefit observed in patients achieving R0 resection. Conversely, suboptimal cytoreduction is associated with significantly worse outcomes, highlighting the importance of appropriate patient selection for primary debulking surgery (PDS). 

A critical and increasingly complex challenge in the management of advanced EOC is determining which patients are suitable candidates for PDS versus those who would derive more benefit from neoadjuvant chemotherapy (NACT) followed by interval debulking surgery (IDS). A large NCDB study comparing PDS and NACT highlighted these challenges but ultimately found improved OS with PDS and R0 resection [6]. While PDS offers the potential for maximal, upfront cytoreduction, it is also associated with increased perioperative morbidity, particularly in patients with extensive disease burden, poor nutritional status, or limited functional reserve. PDS has a greater probability of requiring extensive surgical procedures, such as bowel resection, liver resection, splenectomy, and more—all of which carry inherent risks of their own complications. In contrast, NACT may reduce tumor burden, improve resectability, and decrease surgical morbidity, but concerns remain regarding potential impacts on long-term survival and the development of chemoresistance [7]. Two landmark trials, CHORUS and SCORPION, showed noninferiority of NACT and IDS as compared to PDS in 2015 and 2020, respectively [8,9]. However, the results have been criticized for their limitations. Notably, the lower-than-expected rates of complete resection in these studies potentially underestimated the benefit of PDS. A subsequent randomized Phase III trial failed to reproduce the noninferiority of NACT [10]. The TRUST trial (Trial of Radical Upfront Surgical Therapy in advanced ovarian cancer) is an active, international, multi-center randomized controlled trial aimed to evaluate overall survival in patients with advanced EOC randomized to PDS or NACT followed by IDS [11]. While awaiting final results of this study, the current standard of care remains PDS when clinically appropriate, and optimizing patient selection remains critical.

Traditional preoperative assessment relies on a combination of clinical examination, serum tumor markers such as CA-125, and cross-sectional imaging, most commonly computed tomography (CT). However, these modalities have limited accuracy in predicting the feasibility of optimal cytoreduction. Imaging may underestimate disease in critical areas such as the small bowel mesentery, diaphragmatic surfaces, and porta hepatis, all of which can significantly impact resectability. As a result, a substantial proportion of patients—ranging from 25% to 62% in prior studies—undergo PDS only to have suboptimal debulking, thereby incurring surgical risk without corresponding survival benefit [12].

Initial investigations into CT-based prediction models for cytoreductive surgery showed considerable promise. In 2000, Bristow et al. published findings of a CT-based predictive model achieving impressive results—93% accuracy and 100% sensitivity for predicting suboptimal cytoreduction [13]. Unfortunately, subsequent studies failed to reproduce these encouraging results [14]. Of note, the majority of studies evaluating imaging-based prediction models have been retrospective cohorts with relatively small sample sizes from single institutions. These study designs are subject to several biases, and the findings suggest that CT-based prediction models may be beneficial on an institutional-specific basis. Factors beyond imaging characteristics—such as varying institutional protocols, surgical expertise, and patient selection criteria—limit the generalizability of CT-based prediction models.

Diffusion-weighted magnetic resonance imaging (DW-MRI) has also been studied in the context of predicting cytoreducibility in advanced EOC [15]. Compared to traditional MRI, DW-MRI has been shown to be superior in staging gynecological cancers, diagnosing peritoneal metastases, and differentiating tumor recurrence versus post-treatment changes [16]. A prospective, single-institution study involving 50 patients with ovarian cancer evaluated the peritoneal cancer index (PCI), which is a scoring system assessing 13 anatomic regions, to assess the ability of DW-MRI to accurately predict tumor burden [17]. Of note, most patients in this study were determined to have low tumor burdens. The researchers demonstrated that PCI scores calculated with DW-MRI had a sensitivity of 0.84 and specificity of 0.89 for complete cytoreduction. A meta-analysis came to similar conclusions across 15 articles, suggesting DW-MRI may be a promising modality for initial evaluation of EOC [18]. However, DW-MRI has not been widely adopted due to the limited evidence and lack of large multi-center trials including diverse patients with widespread disease.

To improve patient selection for PDS, several institutional predictive models and triage algorithms have been developed. The Memorial Sloan Kettering resectability score employs a multifactorial approach. It integrates clinical variables, including age, CA-125 levels, and comorbidity burden, with specific radiographic findings to estimate the likelihood of achieving optimal cytoreduction. Implementation of the algorithm in their population achieved a 76% rate of R0 resections and 94% R1 resections in patients triaged to PDS [19,20]. The Mayo Clinic triage algorithm takes a complementary approach, focusing on identifying patients at high risk for perioperative morbidity and mortality rather than cytoreducibility alone. Similarly to the MSK resectability score, this algorithm incorporates variables such as age, performance status, albumin levels, and specific imaging findings to stratify patients into low-, intermediate-, and high-risk categories for surgical complications, thereby guiding decisions regarding surgical candidacy [21]. While these tools have improved clinical decision-making in their respective populations, they face several implementation factors. Both models require validation in more diverse patient populations to establish generalizability of the algorithms. Importantly, these models require a level of specialized radiological expertise that may not be readily available across all practice settings. Additionally, radiographic interpretations are subject to inter-observer variability, which may limit reproducibility of these triage tools.

Diagnostic laparoscopy has emerged as a valuable adjunct in the preoperative evaluation of advanced EOC [22,23]. This minimally invasive approach allows for direct visualization of intra-abdominal disease distribution, including regions that are difficult to assess accurately with imaging alone. The laparoscopic predictive index value (PIV), introduced by Fagotti et al., assigns weighted scores to intraoperative findings including omental caking, diaphragmatic carcinomatosis, peritoneal carcinomatosis, mesenteric retraction, liver metastasis, stomach infiltration, and bowel infiltration [24]. A high PIV score (greater than or equal to 8) has been shown to correlate strongly with unresectable disease and a low likelihood of achieving optimal cytoreduction, enabling clinicians to avoid futile laparotomy and instead proceed with NACT.

Multiple studies since the one by Fagotti et al. have demonstrated that the use of diagnostic laparoscopy improves patient selection for PDS and reduces rates of suboptimal cytoreduction [25]. One retrospective study comparing laparoscopy to CT alone found significantly lower rates of suboptimal cytoreduction in the group evaluated by laparoscopy (2.0% vs. 11.1%, *p* = 0.023), highlighting the value of directly visualizing disease spread [26]. However, the impact of laparoscopy on long-term oncologic outcomes, particularly overall survival, remains less clearly defined. In the same study by Lee et al., Kaplan–Meier analysis showed no significant differences in survival outcomes between the two groups, despite significant improvement in suboptimal cytoreduction with laparoscopy. This discrepancy underscores the need for further investigation to establish the role of laparoscopic evaluation prior to PDS.

Despite growing evidence supporting its use, the adoption of diagnostic laparoscopy in routine clinical practice appears limited, and national utilization trends have not been fully characterized. Given these considerations, we sought to evaluate the use of diagnostic laparoscopy prior to PDS in patients with advanced EOC using the National Cancer Database (NCDB). Our primary objective was to assess the association between laparoscopy use in patients who underwent PDS and overall survival. Secondary objectives included evaluation of perioperative outcomes and trends in utilization over time. We hypothesized that the use of laparoscopy would be associated with improved survival without increasing surgical morbidity.

## 2. Methods

**Figure 1:**
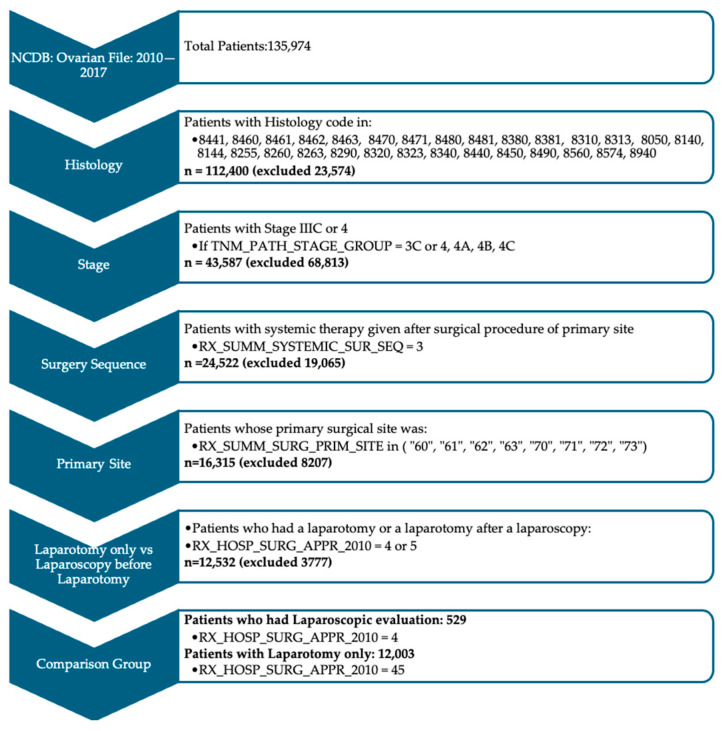
Exclusion cascade and rules.

The NCDB is a clinical database sourced from over 1500 US hospitals accredited by the Commission on Cancer. There were 135,974 patients in the ovarian cancer file who had a diagnosis code of C569. We identified 12,532 patients with stage III-IV EOC who underwent PDS between January 2010 and December 2017. Patients who received NACT prior to IDS were excluded from this study. The patients were divided into two groups for statistical analysis: cases that were initiated with diagnostic laparoscopy, and cases that were initiated with laparotomy alone. Please see Figure 1.

The primary outcome was median OS using the Kaplan–Meier method and was compared across groups using a log-rank test. A significance level of alpha = 0.05 was applied. Other surgical outcomes, including rate of gross residual tumors, extensive surgery, length of hospital stay, readmission within 30 days, and 90-day mortality were analyzed and compared between the two groups using univariate analysis. Age and length-of-stay were summarized with median and IQR and compared across groups using a Wilcoxon sign rank test. Overall survival was summarized with median and IQR and compared across groups using a log-rank test. All other variables were summarized with frequency and percentage and compared across groups using a chi-square test or Fisher’s exact test, where appropriate. Missing data were excluded listwise, and no sensitivity analyses were conducted.

SPSS Version 29.0 (IBM Corp, Armonk, NY, USA) and Joinpoint were used to calculate statistics and surgical trends over time. Due to the de-identified nature of the database, the research was Institutional Review Board exempt.

This paper is an expanded version of an abstracted entitled “Utilization of laparoscopy prior to laparotomy in primary debulking surgery for advanced epithelial ovarian cancer: A National Cancer Database Analysis” which was presented at SGO’s Annual Meeting on Women’s Cancer 2025, Seattle, WA, 14–17 March 2025 [27].

## 3. Results

A total of 12,532 patients with stage IIIC and IV epithelial ovarian cancer who underwent primary debulking surgery between 2010 and 2017 met the inclusion criteria. Of these, 529 patients (4.2%) underwent diagnostic laparoscopy prior to laparotomy, while 12,003 patients (95.8%) underwent direct laparotomy.

Baseline demographic and clinical characteristics were comparable between the two groups. There were no statistically significant differences in age, race, ethnicity, insurance status, or Charlson–Deyo comorbidity scores (Table 1). However, the group who underwent direct laparotomy had a greater proportion of stage IV disease (25.2% vs. 21.4%, *p* = 0.049), which could affect OS analysis. Overall, within the limits of available variables, patients selected for laparoscopy were broadly similar to those undergoing direct laparotomy. This helps limit concerns regarding potential selection bias, although unmeasured confounders may still influence selection.

**Figure 2:**
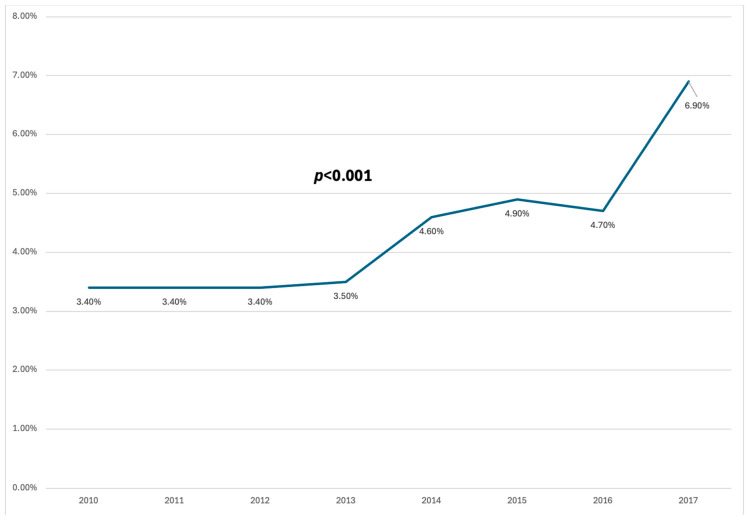
Trends in Laparoscopy Use Prior to Laparotomy.

Temporal trend analysis demonstrated a statistically significant increase in the utilization of diagnostic laparoscopy over the study period. In 2010, 3.4% of PDS cases were initiated with laparoscopy, increasing to 6.9% by 2017 (*p* < 0.001) [Figure 2]. Although this represents a relative doubling in utilization, the overall rate remained low, indicating that laparoscopy has not yet been widely incorporated into standard practice across the United States. However, it is important to note that this study does not capture those cases that were initiated with laparoscopy and then triaged to NACT. Therefore, the overall rate of laparoscopy use may be underestimated.

**Figure 3:**
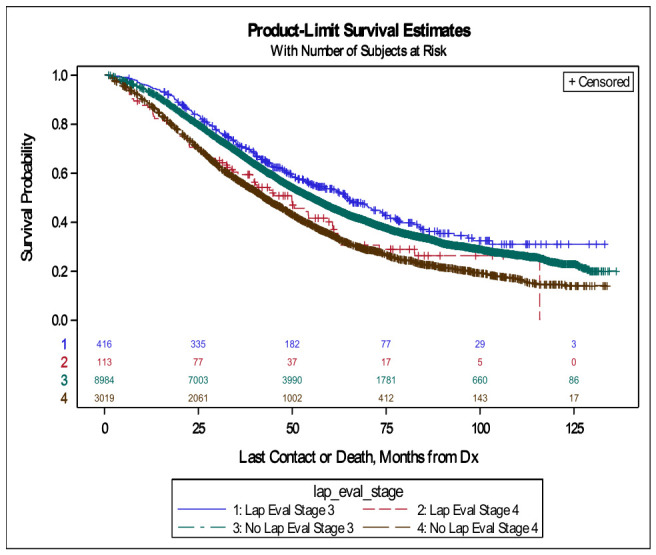
Kaplan-Meier Curves for OS.

With respect to the primary outcome, laparoscopy prior to PDS was associated with longer OS compared to those who underwent direct laparotomy. Median overall survival in the patients who underwent laparoscopy prior to PDS was 60.8 months (95% CI: 54.3–67.2), compared to 51.6 months (95% CI: 50.5–52.7) in the non-laparoscopy group (*p* = 0.012). Kaplan–Meier survival analysis demonstrated early and sustained separation of survival curves, favoring the laparoscopy cohort (Figure 3). Of note, these findings are unadjusted observations.

Given the uneven distribution of stage III and stage IV disease between the groups, stage-stratified analysis was conducted. Longer OS was associated with laparoscopy prior to PDS among patients with stage IIIC disease. In this subgroup, median overall survival was 64.5 months in the laparoscopy group compared to 55.2 months in the non-laparoscopy group (*p* = 0.026). It is again important to note that this association should be interpreted in the context of patients who underwent a diagnostic laparoscopy prior to laparotomy in PDS, as those who were triaged to NACT were not included in analysis. Among patients with stage IV disease, the difference in median overall survival (48.8 vs. 42.7 months) did not reach statistical significance (*p* = 0.47), suggesting that the observed benefit of laparoscopy may be limited in the setting of distant metastatic disease.

Surgical outcomes were similar between groups. The rate of complete gross resection (R0) was 36.5% in the laparoscopy group and 35.2% in the non-laparoscopy group (*p* = 0.553). Rates of extensive surgery, defined by the performance of additional complex procedures beyond total abdominal hysterectomy, bilateral salpingo-oophorectomy, omentectomy, and pelvic and para-aortic lymphadenectomy, were also comparable (52.7% vs. 55.3%, *p* = 0.24). These findings indicate that the use of laparoscopy did not significantly alter the aggressiveness of surgical intervention once laparotomy was undertaken.

Comparison of postoperative outcomes further supported the safety of laparoscopy. There were no statistically significant differences in 90-day mortality rates between the two groups (0.8% vs. 1.7%, *p* = 0.106) or 30-day readmission rates (8.7% vs. 7.8%, *p* = 0.435), Although length of hospital stay was found to be statistically significant (*p* < 0.001), both groups were found to have a median length of stay of 6 days, making this difference unlikely to be clinically significant. These finds suggest that the addition of laparoscopy did not increase perioperative morbidity or healthcare utilization.

## 4. Discussion

In this large, national cohort study, we found that the use of diagnostic laparoscopy prior to primary debulking surgery was associated with significantly longer overall survival among patients with stage IIIC epithelial ovarian cancer. Importantly, this difference was not associated with an increase in perioperative morbidity or mortality, supporting the potential clinical value of this approach. Of note, this interpretation is limited, as the impact of laparoscopy on patients who were triaged to NACT is not addressed in this study.

The survival difference observed in the laparoscopy group is most likely attributable to improved patient selection, rather than the laparoscopic approach itself. Diagnostic laparoscopy enables direct visualization of intra-abdominal disease distribution, providing a level of accuracy that surpasses conventional imaging modalities. By identifying patients with unresectable disease prior to laparotomy, clinicians can avoid non-therapeutic surgical interventions and instead direct these patients toward neoadjuvant chemotherapy. Consequently, patients who proceed to PDS following laparoscopic evaluation are more likely to achieve meaningful cytoreduction, which is strongly associated with improved survival outcomes.

Although prior studies have demonstrated that laparoscopy reduces rates of suboptimal cytoreduction, we did not observe a significant difference in R0 resection rates between groups. This finding should be interpreted with caution, as the NCDB does not capture R1 resections or provide detailed information on residual disease. It is plausible that laparoscopy prior to laparotomy increases the overall rate of optimal cytoreduction (R0 + R1), even if R0 rates alone remain unchanged. Additionally, variations in surgical technique, institutional practices, and surgeon expertise may influence resection outcomes and obscure differences between groups.

The absence of a significant OS difference in stage IV patients is an important observation. In this population, outcomes are largely driven by systemic disease burden and the presence of distant metastases, which may not be amenable to surgical resection. As such, the ability of laparoscopy to influence management decisions and improve outcomes may be limited. This finding highlights the need for individualized treatment strategies based on disease stage and distribution. Additionally, the observed survival difference could also be reflective of unmeasured variables between the groups given the limitations of a database study. Therefore, these findings should be interpreted with caution.

Although this is a hypothesis-generating study, the findings are consistent with prior literature suggesting that laparoscopy is a valuable tool in the management of advanced EOC. Randomized and prospective studies have demonstrated that laparoscopic assessment can reduce unnecessary laparotomies and improve rates of optimal cytoreduction. However, few studies have evaluated its impact on overall survival at a population level. By leveraging a large national database, our study adds valuable insight to a clinically important, potentially practice-changing hypothesis.

Despite these apparent advantages, the utilization of diagnostic laparoscopy remains low. Even by 2017, fewer than 7% of PDS cases were initiated laparoscopically. While this rate does not account for the laparoscopic cases that were triaged to NACT, it is still lower than expected. Several factors may contribute to this underutilization, including variability in surgeon training, lack of standardized protocols, concerns regarding additional operative time, and differences in institutional resources. Additionally, some clinicians may rely primarily on imaging and clinical judgment, particularly in high-volume centers with experienced surgical teams.

The potential risks associated with laparoscopy, such as port-site metastases and influence of insufflation on disease spread, have also been cited as a barrier to adoption. While earlier studies reported relatively high rates of port-site involvement, more recent evidence suggests that these events are uncommon and do not significantly impact overall survival [28,29]. The incidence of port-site metastases has been cited to be as low as 0.26–2.3% [29]. Our findings further support the oncologic safety of laparoscopy, as no adverse survival outcomes were associated with its use.

From a health systems perspective, increasing the use of diagnostic laparoscopy may have important implications. By improving patient selection and reducing unnecessary laparotomies, laparoscopy has the potential to yield significant benefits. Avoiding futile laparotomies in patients unlikely to achieve optimal cytoreduction can lead to decreased healthcare costs and optimized resource utilization. The morbidity associated with an unsuccessful debulking surgery, including prolonged hospital stays, increased resource utilization, and management of postoperative complications, can be a substantial financial burden. Sparing these patients the risks of an unsuccessful laparotomy in favor of proceeding with NACT helps mitigate these costs in addition to minimizing patient morbidity. However, achieving broader use of laparoscopy will likely require targeted efforts in surgeon education, development of standardized guidelines, and integration into multidisciplinary care pathways.

This study has several notable limitations. As a retrospective analysis of a large database, it is subject to inherent biases, including selection bias and unmeasured confounding. Although baseline characteristics were similar between groups, important factors that influence the decision to proceed with PDS or NACT such as tumor distribution, surgical complexity, performance and nutritional status, and surgeon experience are not captured in the NCDB. Additionally, the database lacks information on progression-free survival, chemotherapy regimens, and detailed operative findings, limiting the scope of analysis.

Another limitation is that the NCDB includes only Commission on Cancer-accredited institutions, which may not fully represent all practice settings. Certain patient populations, including those from rural areas or underrepresented minority groups, may be underrepresented in the data set, limiting the generalizability of these results [30]. Finally, our analysis does not include patients who underwent diagnostic laparoscopy but were subsequently triaged to neoadjuvant chemotherapy rather than PDS, potentially underestimating the overall impact of laparoscopy on treatment decision-making.

Despite these limitations, our study provides important insights into the role of diagnostic laparoscopy in advanced EOC. Our findings potentially support its use as a tool for improving patient selection for PDS. However, future research should focus on prospective studies to further validate these findings and to better define the optimal role of laparoscopy in treatment algorithms. Additionally, efforts to standardize laparoscopic scoring systems and integrate them into clinical practice may help maximize their utility. As the field continues to evolve, incorporating minimally invasive approaches into the management of advanced ovarian cancer may play an increasingly important role in improving patient outcomes.

## 5. Conclusions

During our study period, the use of laparoscopy prior to PDS for advanced EOC increased from 3.4% to 6.9%. A statistically significant OS difference was observed with laparoscopy use in the subgroup of patients with stage IIIC disease who proceeded with PDS, but not in patients with stage IV disease (64.5 months vs. 55.2 months, *p* = 0.026). Despite the inherent limitations of a database study, these findings support prior studies demonstrating the potential benefit of laparoscopic evaluation of advanced EOC. Additionally, there were no adverse outcomes associated with laparoscopy use. Future prospective studies are necessary to validate these findings and optimize surgical decision-making and the role of laparoscopy in advanced EOC.

## Figures and Tables

**Table 1:** Patient Characteristics and Surgical Outcomes.

		Laparotomy Only (n = 12,003)	Laparoscopy Before Laparotomy (n = 529)	*p*-Value
Age at Diagnosis		61 (53–69) ^1^	62 (53–69) ^1^	0.911
Race	White	10,514 (87.6%)	454 (85.8%)	0.117
	Black	834 (6.9%)	38 (7.2%)	
	Asian/Pacific Islander	444 (3.7%)	30 (5.7%)	
	Other	142 (1.2%)	3 (0.6%)	
	Unknown	69 (0.6%)	4 (0.8%)	
Ethnicity	Not Hispanic	11,032 (91.9%)	488 (92.2%)	0.26
	Hispanic/Presumed Hispanic	690 (5.7%)	34 (6.4%)	
	Unknown	281 (2.3%)	7 (1.3%)	
Insurance	No Insurance	440 (3.7%)	9 (1.7%)	0.075
	Private Insurance	6072 (50.6%)	279 (52.7%)	
	Medicaid/Medicare/Other Public	5344 (44.5%)	232 (43.9%)	
	Unknown	147 (1.2%)	9 (1.7%)	
Stage	IIIC	8984 (74.8%)	416 (78.6%)	0.049
	IV	3019 (25.2%)	113 (21.4%)	
Charlson–Deyo Score ≥ 1		2633 (21.9%)	119 (22.5%)	0.761
No Gross Residual		4228 (35.2%)	193 (36.5%)	0.553
Extensive Surgery		6642 (55.3%)	279 (52.7%)	0.24
Length of Stay		6 (5–9)	6 (4–8)	<0.001
Readmission within 30 days		932 (7.8%)	46 (8.7%)	0.435
90 Day Mortality		200 (1.7%)	4 (0.8%)	0.106
Overall Survival in Months		51.6 (50.5–52.7) ^2^	60.8 (54.3–67.2) ^2^	0.012

^1^ Median (IQR); ^2^ Median (Confidence Interval).

## Data Availability

The data presented in this study are openly available in the national cancer database (NCDB), https://www.facs.org/quality-programs/cancer-programs/national-cancer-database, accessed on 18 September 2024.
